# Isolated central nervous system relapse of chronic myeloid leukemia after allogeneic hematopoietic stem cell transplantation

**DOI:** 10.1186/1471-2326-12-9

**Published:** 2012-08-07

**Authors:** Mary Fuchs, Mike Reinhöfer, Andreas Ragoschke-Schumm, Herbert G Sayer, Klas Böer, Otto W Witte, Andreas Hochhaus, Hubertus Axer

**Affiliations:** 1 Hans Berger Department of Neurology, Jena University Hospital, Erlanger Allee 101, D-07747 Jena, Germany; 2 Department of Clinical Chemistry and Laboratory Diagnostics, Jena University Hospital, Erlanger Allee 101, D-07747 Jena, Germany; 3 Institute of Diagnostic and Interventional Radiology, Department of Neuroradiology, Friedrich-Schiller-University Jena, Erlanger Allee 101, D-07747 Jena, Germany; 4 Department for Hematology and Oncology, Clinic for Internal Medicine II, Jena University Hospital, D-07747 Jena, Germany

**Keywords:** Chronic myeloid leukemia, CNS relapse, BCR-ABL

## Abstract

**Background:**

This case report highlights the relevance of quantifying the BCR-ABL gene in cerebrospinal fluid of patients with suspected relapse of chronic myeloid leukemia in the central nervous system.

**Case presentation:**

We report on a female patient with isolated central nervous system relapse of chronic myeloid leukemia (CML) during peripheral remission after allogeneic hematopoietic stem cell transplantation. The patient showed a progressive cognitive decline as the main symptom. MRI revealed a hydrocephalus and an increase in cell count in the cerebrospinal fluid (CSF) with around 50% immature blasts in the differential count. A highly elevated BCR-ABL/ ABL ratio was detected in the CSF, whilst the ratio for peripheral blood and bone marrow was not altered. On treatment of the malresorptive hydrocephalus with shunt surgery, the patient showed an initial cognitive improvement, followed by a secondary deterioration. At this time, the cranial MRI showed leukemic infiltration of lateral ventricles walls. Hence, intrathecal administration of cytarabine, methotrexate, and dexamethasone was initiated, which caused a significant decrease of cells in the CSF. Soon after, the patient demonstrated significant cognitive improvement with a good participation in daily activities. At a later time point, after the patient had lost the major molecular response of CML, therapy with dasatinib was initiated. In a further follow-up, the patient was neurologically and hematologically stable.

**Conclusions:**

In patients with treated CML, the rare case of an isolated CNS blast crisis has to be taken into account if neurological symptoms evolve. The analysis of BCR-ABL in the CSF is a further option for the reliable detection of primary isolated relapse of CML in these patients.

## Background

Chronic myeloid leukemia (CML) is a myeloproliferative disorder characterized by the presence of the Philadelphia chromosome which is caused by a reciprocal translocation between chromosome 9 and 22. The result is a fusion gene from a part of the *BCR* (breakpoint cluster region) gene from chromosome 22 and the *Abl1* gene from chromosome 9 [1]. Further, extramedullary blast crisis is a known complication of CML. However, the central nervous system as an isolated site of extramedullary blast crisis is rare [2].

We report on a 64 year-old woman with CML in remission who developed an isolated central nervous system relapse after an unrelated one antigen mismatched allogeneic hematopoietic stem cell transplantation.

## Case presentation

CML was first diagnosed in January 2005 with a blast crisis. The patient was subsequently treated with imatinib. In November 2005, therapy was changed to cytosine arabinoside and mitoxantrone followed by hydroyurea due to a second blast crisis. Since February 2006, the second generation tyrosine-kinase inhibitor (TKI) dasatinib induced a hematological remission (chronic phase) until a one antigen mismatched (C-allele locus) unrelated allogeneic hematopoietic stem cell transplantation (SCT) was performed in May 2006. After SCT, she developed a series of epileptic seizures owing to posterior reversible encephalopathy syndrome (PRES) and developed severe critical illness polyneuropathy. At this time point, the analysis of the CSF was normal (1 cell/μl, total protein 355 mg/l) pointing neither to inflammation nor to a relapse. After initial severe tetraplegia, she reconstituted during intensive rehabilitation therapy and could use her arms independently, but did not regain her ability to walk. Up to November 2007, the patient received immunosuppressive therapy with ciclosporine and low dose prednisolone was administered until May 2008 because of a mild hepatic graft-versus-host disease. Cognition remained unimpaired. In all follow-up hematological visits after transplant, CML was regarded to be in remission (major cytogenetic and major molecular).

**Figure 1:**
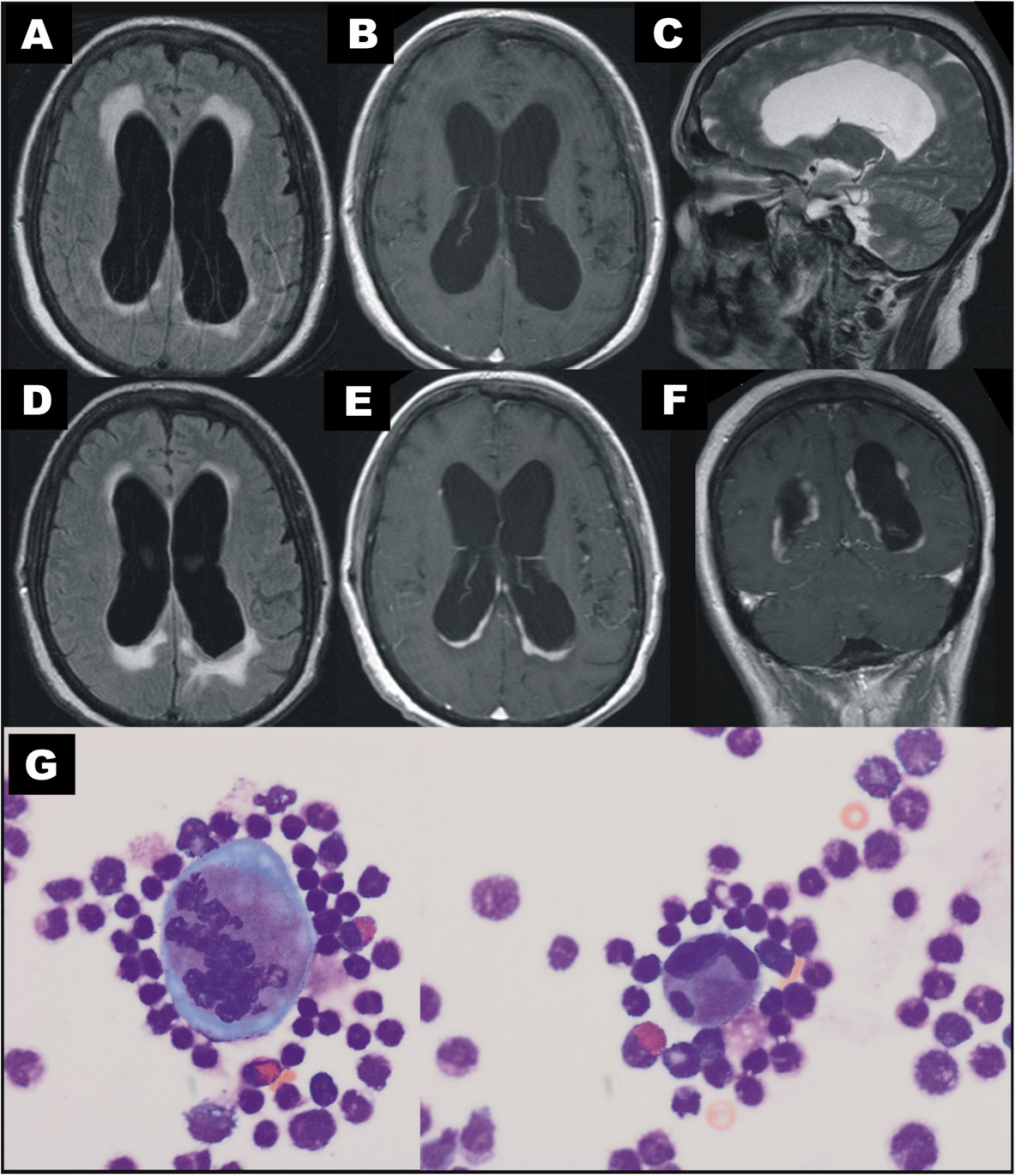
**MR and CSF: First MR showed a hydrocephalic enlargement of the lateral ventricles (A FLAIR, B Gd-enhanced T1w, C T2w), while gadolinium-enhanced T1w did not show significant enhancement (B).** Follow-up MR after secondary deterioration of the patient: showed leukemic infiltrations of the lateral ventricles walls (**D** FLAIR, **E** and **F** Gd enhanced T1w). CSF (**G**) revealed immature blasts with a pathological plasma-nucleus relation and basophilia of cytoplasm.

In November 2008, a progressive cognitive decline within a period of 6 months was noticed which led to a neurological consultation. The patient was mutistic and apathetic showing psychomotorical impairment and pathologically inadequate laughter. Moreover, orientation regarding time and place was impaired, but spastic tetraplegia was unchanged. MRI revealed a hydrocephalus with signs of high brain pressure (Figure 1 A-C). A lumbar puncture showed an elevated total cell count (389 cells /μl) and total protein (1154 mg/l) with an increased pressure of 26.5 cm H_2_O. Thus, 30 ml of CSF was drained leading to a significant cognitive improvement.

CSF microbiology excluded an infectious cause of the pleocytosis. In the differential count of CSF, about 50% immature blasts were counted and 65% myeloid precursor cells (CD7/CD33 double positive) were detected by FACS-analysis. However, the peripheral blood differential count was normal and did not point to a systemic hematological relapse of CML. The BCR-ABL/ ABL ratio (real time PCR) in CSF was 61.44% (and 0.0025% in the bone marrow).

The malresorptive hydrocephalus was at first treated with shunt surgery since the hydrocephalus was thought to be the major pathophysiologic factor causing cognitive decline in the patient. Following surgery, the CSF cell count fell to 66 cells/μl. However, after initial cognitive improvement, the patient showed a secondary deterioration. At this time point, leukemic infiltration in the lateral ventricles walls was detected by cranial MRI (Figure 1 D-F). Total cell and total protein counts were 1024 cells/μl and 2923 mg/l, respectively, and 66% of the cells were CD7/CD33 double positive by FACS-analysis. In the CSF, differential count megakaryocytes, immature eosinophils and blast cells were noted. (Figure 1 G). The BCR-ABL/ABL ratio (real time PCR) in the bone marrow had increased from 0.0025% in the previous analysis to 0.07%.

Triple intrathecal therapy consisting of cytarabine (40 mg), methotrexate (10 mg), and dexamethasone (4 mg) was initiated, as described in the literature for cases of CNS relapse [2-4]. Intrathecal chemotherapy was administered twice and caused a significant decrease of total cell count and protein in the CSF (11 cells/μl, 1254 mg/l protein). Myeloid precursor cells were reduced to 19% by FACS-analysis. During follow-up examinations, cognition of the patient was much improved and she showed good participation in daily activities.

Therapy with dasatinib was begun after initial intrathecal chemotherapy, due to its ability to cross the blood–brain barrier [5] and because the major molecular response was lost in the consecutive bone marrow aspiration in February 2009 (BCR-ABL/ ABL ratio (real time PCR): 0.24%). Since then, the patient has remained neurologically stable with subjective improvement of her impaired daily activities. No hematological relapse occurred during further patient follow up and continuous dasatinib therapy. However, the patient was diagnosed with advanced vulva carcinoma in May 2011 and died in December of the same year.

## Discussion

BCR-ABL transcripts are regarded as early markers for a hematological relapse of CML [1]. Oral tyrosine kinase inhibitors such as imatinib inhibit BCR-ABL-tyrosine kinase. However, imatinib does not penetrate the blood–brain-barrier so that isolated CNS blast crises have been described in several cases [2-4]. Single cases of isolated CNS blast crises have also been depicted, for example, in one patient under dasatinib [6], a second generation TKI with an improved penetration of the blood–brain-barrier, and after allogeneic hematopoietic stem cell transplantation in two cases [7]. Thus, the CNS has to be regarded as a sanctuary site of relapse. This may generally be caused by decreased levels of the drugs being found in the CNS [2]. The history of CNS involvement before hematopoietic stem cell transplantation has been identified as significant predictors for CNS relapse after hematopoietic stem cell transplantation [7].

The isolated CNS relapse in our patient was detected due to the presence of blasts and other myeloid precursor cells in the CSF as well as increased BCR-ABL quantities over time, while blood and bone marrow did not reveal such abnormalities at that time. The increase of cells in CNS led to a malresorptive hydrocephalus which had to be treated with shunt surgery. The treatment of isolated CNS relapse comprises of intrathecal chemotherapy and/or cranial irradiation [7]. Donor lymphocyte infusion (DLI) was not considered as the first therapeutic option in this case, due to the dramatical clinical course. Intrathecal chemotherapy led to a significant reduction of CSF cells indicating successful control of CNS disease besides the neurological improvement, so that additional radiotherapy was not considered.

## Conclusion

In patients with treated CML, the rare case of an isolated CNS blast crisis has to be taken into account if neurological symptoms evolve. Lumbar puncture is obligatory and isolated CNS relapse of leukaemia can be controlled. The analysis of BCR-ABL in the CSF is a further option either for reliable detection of isolated relapse or as the first sign of a consecutive hematological relapse of CML.

## Consent

Written informed consent was obtained from the patient for publication of this case report and accompanying images. A copy of the written consent is available for review by the Editor in Chief of this journal.

## Abbreviations

CML: Chronic myeloid leukemia; CSF: Cerebrospinal fluid; CNS: Central nervous system; FACS: Fluorescence-activated cell sorting; BCR-ABL: Oncogenic BCR-ABL gene fusion; TKI: Tyrosine-kinase inhibitor.

## Competing interests

The authors declare that they have no competing interests.

## Authors’ contributions

MF and HA drafted the first manuscript and made a contribution to acquisition and interpretation of data. MF, OW and HA are treating neurologists of the patient and made a contribution to acquisition and interpretation of data. HGS and AH are treating hematologists of the patient and had a role in data acquisition and interpretation. MR and KB performed the laboratory analyses and contributed to acquisition and interpretation of data. ARS is neuroradiologist and performed and analyzed the MRI data. All authors revised the manuscript and approved the final manuscript. We thank Nasim Kroegel for her help in language editing.

## Pre-publication history

The pre-publication history for this paper can be accessed here:

http://www.biomedcentral.com/1471-2326/12/9/prepub
